# Class II malocclusion with accentuated occlusal plane inclination corrected with miniplate: a case report

**DOI:** 10.1590/2177-6709.21.3.094-103.oar

**Published:** 2016

**Authors:** Marcel Marchiori Farret, Milton M. Benitez Farret

**Affiliations:** 1 Professor, post-graduation courses, Specialization in Orthodontics, Centro de Estudos Odontológicos Meridional (CEOM), Passo Fundo, Rio Grande do Sul, Brazil; and Fundação para Reabilitação das Deformidades Crânio-Faciais (FUNDEF), Lajeado, Rio Grande do Sul, Brazil.; 2 Professor, Universidade Federal de Santa Maria (UFSM), Santa Maria, Rio Grande do Sul, Brazil.

**Keywords:** Angle Class II malocclusion, Orthodontic anchorage procedures, Orthodontic appliance design

## Abstract

**Introduction:**

A canted occlusal plane presents an unesthetic element of the smile. The correction of this asymmetry has been typically considered difficult by orthodontists, as it requires complex mechanics and may sometimes even require orthognathic surgery.

**Objective:**

This paper outlines the case of a 29-year-old woman with Class II malocclusion, pronounced midline deviation and accentuated occlusal plane inclination caused by mandibular deciduous molar ankylosis.

**Methods:**

The patient was treated with a miniplate used to provide anchorage in order to intrude maxillary teeth and extrude mandibular teeth on one side, thus eliminating asymmetry. Class II was corrected on the left side by means of distalization, anchored in the miniplate as well. On the right side, maxillary first premolar was extracted and molar relationship was kept in Class II, while canines were moved to Class I relationship. The patient received implant-prosthetic rehabilitation for maxillary left lateral incisor and mandibular left second premolar.

**Results:**

At the end of treatment, Class II was corrected, midlines were matched and the canted occlusal plane was totally corrected, thereby improving smile function and esthetics.

## RESUMO

**Introdução:**

o plano oclusal inclinado representa um elemento antiestético para o sorriso. A correção dessa assimetria é normalmente considerada difícil pelos ortodontistas, requerendo mecânica complexa e, algumas vezes, até cirurgia ortognática.

**Objetivo:**

esse artigo descreve o caso de uma paciente de 29 anos, portadora de má oclusão de Classe II, com considerável desvio das linhas médias e acentuada inclinação do plano oclusal, causada pela anquilose de molar decíduo inferior.

**Métodos:**

a paciente foi tratada com ancoragem em miniplaca, para promover a intrusão dos dentes superiores e extrusão dos dentes inferiores em um lado, eliminando a assimetria. A Classe II foi corrigida no lado esquerdo por meio de distalização, também ancorada na miniplaca. No lado direito, o primeiro pré-molar superior foi extraído e a relação de molares de Classe II foi mantida, enquanto os caninos foram movidos para relação de Classe I. A paciente recebeu reabilitação por meio de implante e prótese no incisivo lateral superior esquerdo e no segundo pré-molar inferior esquerdo.

**Resultados:**

ao término do tratamento, a Classe II foi corrigida, as linhas médias estavam coincidentes e a inclinação do plano oclusal foi totalmente corrigida, melhorando consideravelmente os aspectos funcionais e estéticos da oclusão.

## INTRODUCTION

Occlusal plane inclination has always represented a challenge for orthodontists.^1^ The common options for treatment included asymmetric mechanics with high-pull headgears, asymmetric bite blocks,^2^ ^,^ ^3^ ^,^ ^4^ or even orthognathic surgery in some cases.^5^ ^,^ ^6^ ^,^ ^7^ In such cases, conventional mechanics require a long time to be performed, and adverse effects are often present, thus compromising and limiting treatment results.^2^ ^,^ ^8^ ^,^ ^9^ Furthermore, patients frequently refuse orthognathic surgery and, as such, all treatment options for a canted occlusal plane have limitations.^10^

The introduction of skeletal anchorage has increased the number of treatment options for these cases.^2^ ^,^ ^8^ ^,^ ^11^ ^,^ ^12^ Mini-implants or miniplates may aid intrusion of a group of teeth, either in the maxillary or mandibular arches, without adverse effects while greatly reducing total treatment time.^9^ ^,^ ^13^ For large asymmetries, it is preferable to use miniplates, owing to the greater stability and success rate obtained with this device in comparison with mini-implants.^2^ ^,^ ^11^ ^,^ ^13^ ^,^ ^14^ ^,^ ^15^

In this paper, correction of occlusal plane inclination by means of skeletal anchorage is discussed. A case is presented in which significant asymmetry was corrected with a miniplate as the anchorage unit.

## CASE REPORT

### Diagnosis and etiology

**Figure 1:**
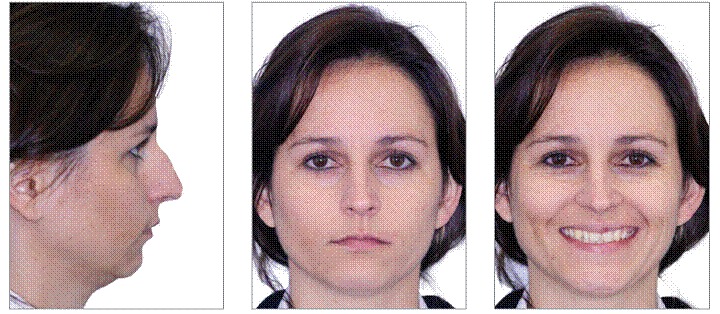
Pretreatment facial photographs.

**Figure 2:**
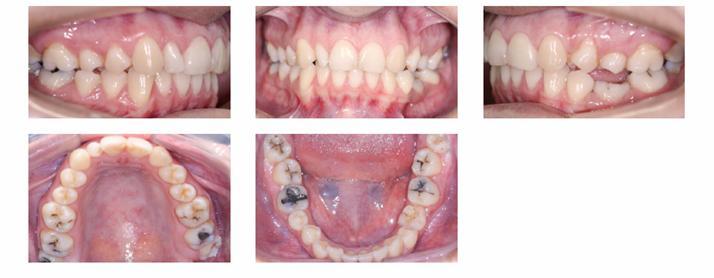
Pretreatment intraoral photographs.

A 29-year-old woman sought orthodontic treatment, complaining about an unesthetic smile due to occlusal plane inclination and midline deviation. This was caused by absence of maxillary left lateral incisor and mandibular left second premolar, with ankylosis of deciduous molar in this region. Facial analysis revealed good symmetry and vertical balance of the facial thirds, a convex profile, and accentuated occlusion plane inclination in a smiling photograph (Fig 1). Intraoral analysis revealed Angle Class II, Division 1 malocclusion, with absence of maxillary left lateral incisor, a peg-shaped maxillary right lateral incisor and the presence of mandibular left deciduous ankylosed second molar, which caused asymmetry on this side in both maxillary and mandibular arches (Figs 2 and ^3^). Maxillary midline was deviated 2 mm to the left while mandibular midline was deviated 2 mm to the right. Panoramic and periapical radiographs confirmed the absence of maxillary lateral incisor and mandibular second premolar and also revealed mandibular teeth greatly inclined towards the ankylosed deciduous molar. Initial lateral cephalogram and cephalometric tracing revealed skeletal Class II malocclusion, with upright maxillary incisors and well-positioned mandibular incisors (Fig 4 and Table 1). 

**Figure 3:**
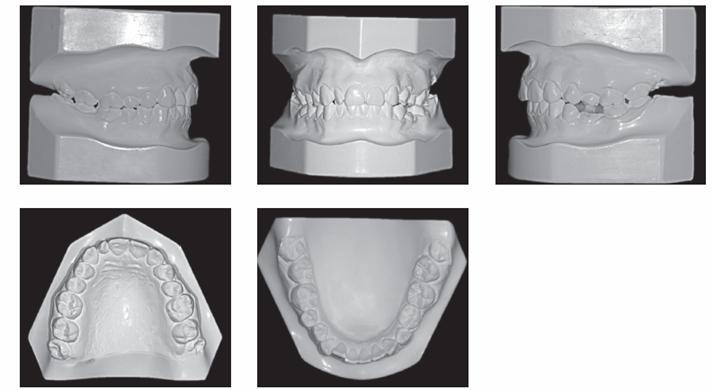
Pretreatment dental casts.

**Figure 4:**
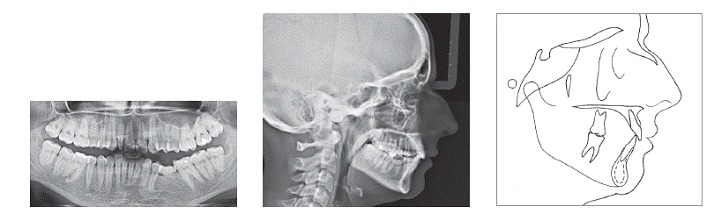
Pretreatment panoramic radiograph, lateral cephalogram and cephalometric tracing.

**Table 1:** Cephalometric measurements.

Measurements	Norms	Initial	Post-treatment
SNA	82°	81	80
SNB	80°	76	78
ANB	2°	5	2
Angle of convexity	0°	10	3
Facial angle	87°	85	87
Y-axis	59°	59	56
SN-GoGn	32°	33	29
1.NA (degrees)	22°	20	30
1-NA (mm)	4mm	3	5
1.NB (degrees)	25°	25	30
1-NB (mm)	4mm	5	6
^- Interincisal angle^	130°	129	116
Upper lip - S-line	0mm	1.5	1.5
Lower lip - S-line	0mm	0.5	1.5
IMPA	90°	97	102
FMA	25°	24	21
FMIA	65°	59	57

### Treatment objectives

The objectives of treatment were as follows:

1. Correct occlusal plane inclination.
2. Obtain molar Class I relationship on the left side and Class II on the right side.
3. Establish canine Class I relationship on both sides.
4. Correct midlines.
5. Extract deciduous molar and replace the tooth with implant-prosthetic rehabilitation.
6. Open space in order to implant a prosthetic rehabilitation of the maxillary left lateral incisor.

### Treatment alternatives

Orthognathic surgery was considered for occlusal plane correction, but the patient refused this option. Therefore, two other alternatives were considered to correct Class II malocclusion and tooth absences. The first option was to extract the maxillary right lateral incisor, replace lateral incisors with canines, and then replace canines with first premolars. This option was rejected in a meeting with the dentist responsible for the final rehabilitation. The dentist believed that the esthetic result would be better with implant-prosthetic rehabilitation of the maxillary lateral incisor, as maxillary canines had large crowns and were too different in color, so as to be used as lateral incisors. The second option was to extract maxillary right first premolar and insert a mini-implant or miniplate on the left side to move the maxillary right dentition posteriorly. This option was rejected by the patient due to longer treatment time required in comparison to that for first premolar extraction to distalize all teeth. Thus, in agreement with the patient and the other dentist, it was decided to correct the occlusal plane by means of a miniplate on the maxillary left side, extract the maxillary right first premolar and open space for rehabilitation of the maxillary left lateral incisor. 

### Treatment progress

**Figure 5:**
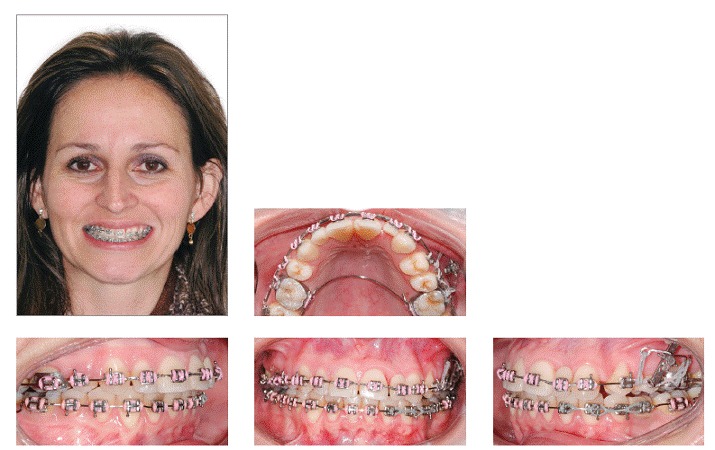
Photographs after the insertion of miniplate and occlusal plane correction onset.

**Figure 6:**
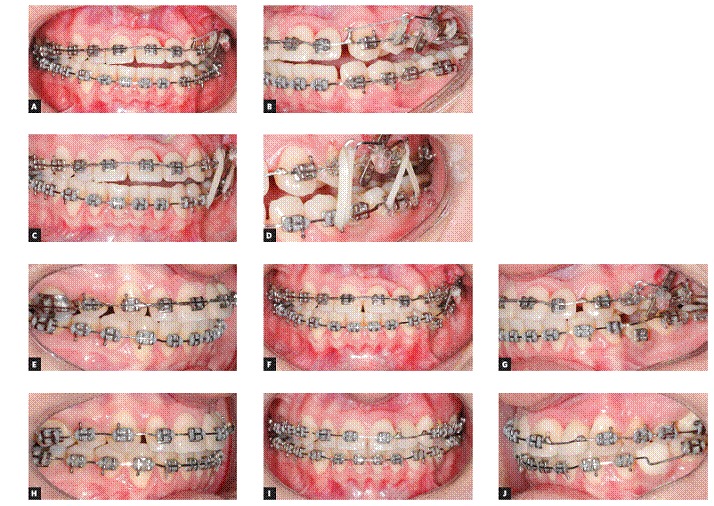
Intraoral mechanic sequence. (A and B) After maxillary right teeth intrusion, (C and D) elastic mechanics employed to extruded mandibular left teeth, (E to G) after mandibular extrusion, (H to J) after miniplate removal and during the finishing procedures.

Treatment began with the bonding of 0.022 × 0.028-in standard Edgewise brackets on both arches, followed by alignment and leveling with 0.012 and 0.014-in Nickel-Titanium archwires and from 0.014-in to 0.020-in stainless steel archwires. Thereafter, maxillary right first premolar and mandibular left second deciduous molar were extracted and maxillary anterior teeth were moved to the right, tooth by tooth, with elastomeric chains, in order to correct maxillary midline and open space, thus allowing the insertion of an implant in the space left by the maxillary left lateral incisor. On the maxillary left side, after correction of premolars rotation, a 2-mm space was created and both premolar and canine were distalized with elastomeric chains to increase the space for implantation of the maxillary left lateral incisor prosthesis and to partially correct Class II. On the mandibular arch, an implant was inserted into the space of the missing premolar to aid mandibular midline correction. That implant was positioned above the proper position, considering that after occlusal plane correction with maxillary intrusion and mandibular extrusion on this side, the implant would be in adequate vertical position. Likewise, the implant was positioned closer to the mandibular left first molar and away from the left first premolar, thereby allowing distalization of mandibular left molars and distalization of mandibular anterior teeth, thus correcting the midline. After that, a miniplate in the shape of an Y was inserted in left zygomatic buttress and used to intrude all maxillary left teeth, with elastics connected to 0.019 × 0.025-in wire segments inserted into a tube and connected to a miniplate, generating a force of 200 g/f each (Fig 5). Furthermore, the miniplate was used to distalize all teeth on the left side, with elastomeric chains connected to a hook welded between the lateral incisor and canine, so as to correct Class II relationship. After correction on the maxillary arch, the mandibular arch was extruded with intermaxillary 1/8-in elastics connected directly to the miniplate and on the mandibular teeth and archwire (Fig 6). In order to allow mandibular teeth extrusion, the mandibular arch was made bypassing the bracket of provisory crown over the implant. At that time, the space for maxillary left lateral incisor was already well defined and the implant was inserted. Maxillary right lateral incisor was provisionally restored with composite resin before appliance debonding, so as to precisely define the spaces on the anterior region. After 34 months of treatment, the appliance was removed. 

### Treatment results

**Figure 7:**
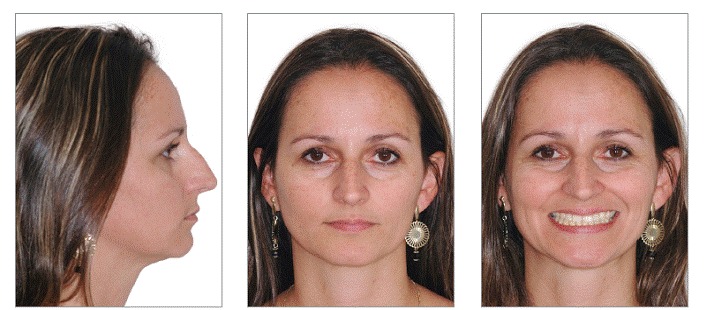
Post-treatment facial photographs.

**Figure 8:**
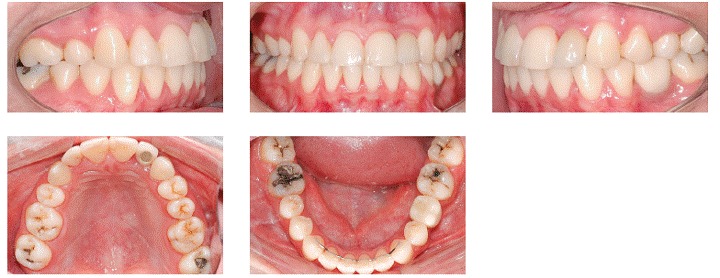
Post-treatment intraoral photographs.

At the end of treatment, we noticed an improvement in smile esthetics due to correction of occlusal plane inclination and because the midlines were coincident with the facial midline (Fig 7). The profile remarkably improved as a result of counterclockwise rotation of the mandible, which reduced convexity, thus increasing the prominence of lips and chin (Fig 7). Intraoral and dental casts analyses revealed that Class I molar relationship on the left side, Class II molar relationship on the right side and Class I canine relationship on both sides were all obtained, with good intercuspation (Figs 8 and ^9^). Panoramic radiograph showed good parallelism among roots, in addition to root resorption on maxillary left central incisor, which will be monitored after treatment. Post-treatment lateral cephalogram, cephalometric tracing and superimposition examinations confirmed accentuated mandibular counterclockwise rotation (Fig 10). Furthermore, maxillary left molars were intruded while mandibular molars were uprighted and extruded. Maxillary and mandibular incisors were proclined after treatment. The patient will be monitored every six months in order to have root resorption and treatment stability controlled. 

**Figure 9:**
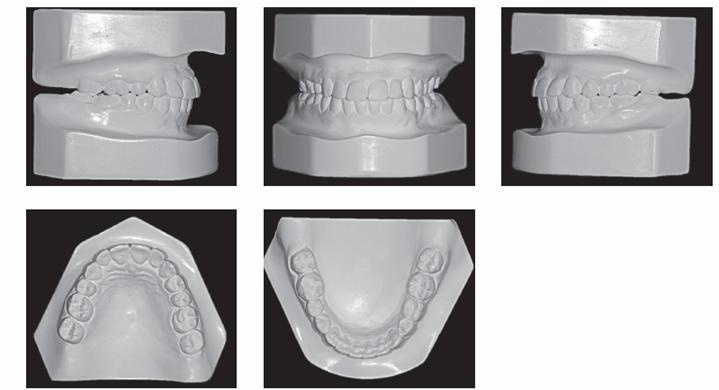
Post-treatment dental casts.

**Figure 10:**
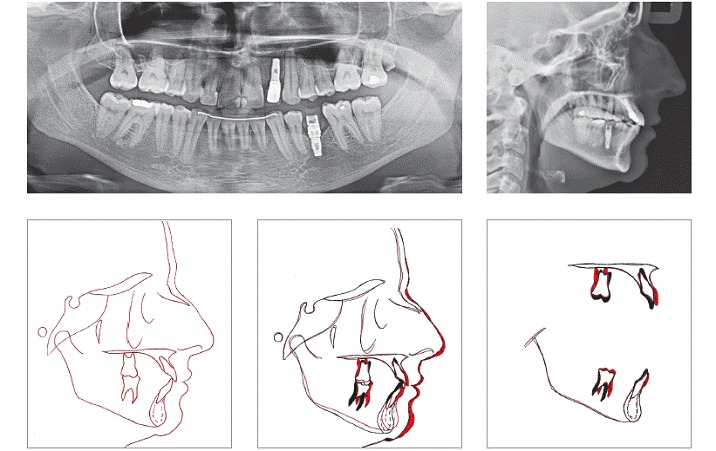
Post-treatment panoramic radiograph, lateral cephalogram, cephalometric tracing, total superimposition, maxillary superimposition and mandibular superimposition.

## DISCUSSION

Occlusal plane inclination is recognized as an asymmetry that impairs smile esthetics.^16^ ^,^ ^17^ Padwa et al^17^ and Pereira et al^18^ studied some variations in occlusal plane inclination and found that as the degree of this asymmetry increases, the perceived attractiveness decreases. According to the authors, one of the reasons may be gingival exposure only on one side. This asymmetry should be corrected either by intrusion on one side, extrusion on the other side or a combination of both, depending on the diagnosis and treatment planning.^8^ Intrusion is directed on the maxillary arch when gingival exposure is accentuated, followed by mandibular extrusion on the same side. Otherwise, when there is no gingival exposure associated with occlusal plane inclination, intrusion must be carried out on one side of the mandibular arch, followed by extrusion on the same side of the maxillary arch, considering that intrusion on the maxillary arch could extremely reduce maxillary teeth exposure, impairing smile esthetics. The combination of both procedures may be used in cases with moderate gingival exposure.^8^ A precise esthetic diagnosis should be performed in these cases, including a series of smile photographs and thorough clinical examination. Frontal cephalograms are also an important tool for diagnosis and are essential, mainly when orthognathic surgery is being considered.^6^ ^,^ ^17^

Traditionally, the treatment options for asymmetries in the occlusal plane have been considered to be major challenges for orthodontists.^1^ Despite the complexity of procedures, surgical approaches have always been considered to be a good option, as they have a reduced treatment time and avoid some adverse effects of conventional orthodontic mechanics.^5^ ^,^ ^6^ ^,^ ^8^ However, the majority of patients refuse orthognathic surgery and treatment must therefore focus on orthodontic camouflage. One option is to use a unilateral bite block, which is another alternative for treatment and may provoke a minor intrusion on the side where it is located and a more significant extrusion on the other side. The limitation of this treatment modality is that it is not possible to attain moderate to high intrusion movements with these devices, in addition to the possibility of developing temporomandibular disorders after long periods of use. Other option consists in using an asymmetric high-pull headgear; however, it depends on patient's compliance and has limited results even after long periods of use. The main reason for that is because the force between both sides cannot be very different in order to prevent displacement of occipital strap. 

Skeletal anchorage appeared a few years ago as an excellent alternative for the treatment of asymmetries. It has no adverse effects on mechanics and does not rely on patient's compliance, meaning that treatment is more predictable and reliable.^11^ ^,^ ^19^Specifically for occlusal plane inclination, mini-implants may be the favored option for cases of minor discrepancies and two mini-implants should be preferably used in order to increase retention. Other problems related to mini-implants is the risk of root contact during treatment, as the intrusion movement is performed towards the mini-implant.^20^ For these reasons, miniplates may be a better option for the treatment of vertical asymmetries on the occlusal plane, delivering an excellent capacity to intrude a group of teeth without the risk of coming into contact with any of the roots during treatment.^3^ ^,^ ^4^ ^,^ ^11^ ^,^ ^15^However, the disadvantage of miniplates is the need for two invasive surgical procedures to insert and remove the device, the reason why patients sometimes refuse miniplates.^15^

Root resorption may be a consequence of orthodontic treatment. Constant forces usually provoke higher root resorption in comparison with interrupt forces. Other authors agree with it and according to them it happens because the pause in force allows the resorbed cementum to heal and prevents further resorption.^21^ ^,^ ^22^ ^,^ ^23^ Furthermore, intrusion movement is one of the main causes of resorption as well.^24^ In the case described herein, the maxillary arch was intruded on the left side with constant forces delivered by elastics connected to the miniplate, which probably caused some root resorption on maxillary anterior teeth, which was more accentuated on the left side. After the end of active orthodontic treatment, root resorption tends to stop;^25^ ^,^ ^26^ therefore, the patient will be monitored every six months to check whether resorption has indeed stopped. 

Unfortunately, there are no studies in the literature that have analyzed the long-term stability of occlusal plane inclination correction by means of skeletal anchorage. The magnitude of orthodontic movement obtained with miniplates is remarkably higher than that obtained in the past with conventional mechanics. In order to avoid relapses, it is recommended that the appliance is stabilized for at least six months after correction, allowing for complete bone remodeling and reorganization of fibers. The retention protocol is the same as that usually used in other cases, with a 3 × 3 mandibular bonded retainer and a wraparound removable appliance on the maxillary arch. The patient must be monitored for a long period of time in order to identify any relapse and intercept or treat it. 

## CONCLUSION

The literature and case presented herein demonstrate that miniplates are a reliable device for the correction of occlusal plane inclination, eliminating the need for orthognatic surgery in some cases and reducing the complexity of orthodontic mechanics.
